# Mediterranean Diet for Primary and Secondary Prevention of Cardiovascular Disease and Mortality: An Updated Systematic Review

**DOI:** 10.3390/nu15153356

**Published:** 2023-07-28

**Authors:** Ana Laffond, Cristina Rivera-Picón, Pedro Manuel Rodríguez-Muñoz, Raúl Juárez-Vela, Regina Ruiz de Viñaspre-Hernández, Noelia Navas-Echazarreta, Juan Luis Sánchez-González

**Affiliations:** 1 Department of Medicine, University Hospital of Salamanca, 37007 Salamanca, Spain; anaelaffond@gmail.com; 2 Faculty of Health Sciences, Nursing, Pontifical University of Salamanca, 37002 Salamanca, Spain; criverapi@upsa.es; 3 Faculty of Nursing and Physiotherapy, University of Salamanca, 37008 Salamanca, Spain; pedromrodriguez@usal.es (P.M.R.-M.); juanluissanchez@usal.es (J.L.S.-G.); 4 Research Group GRUPAC, Nursing Department, Faculty of Heatlh Sciences, University of La Rioja, 26004 Logrono, Spain; raul.juarez@unirioja.es (R.J.-V.); noelia.navas@unirioja.es (N.N.-E.)

**Keywords:** Mediterranean diet, cardiovascular disease, mortality, systematic review, prevention

## Abstract

Cardiovascular diseases (CVDs) are currently the leading cause of mortality worldwide, with coronary heart disease being the primary cause. The Mediterranean Diet (MD) has been highlighted for its potential in providing greater protection against CVDs. This study aims to present an updated systematic review that examines the impact of MD on mortality and CVDs, both in the general population and in patients with a prior CVD, while also considering the potential influence of gender. We conducted a systematic review. After the selection process, 24 studies met the inclusion criteria. The findings from these studies consistently demonstrate that higher adherence to the MD is associated with a reduced risk of overall mortality, both in the general population and in patients with previous CVDs. Moreover, evidence suggests that following this dietary pattern likely decreases the risk of CVDs such as heart attacks, various types of coronary artery disease, stroke, and cardiovascular mortality. While some studies have identified differences in the benefits of the MD between men and women, it is important to note that these disparities may be attributed to lower event rates and a generally lower cardiovascular risk profile in women. Thus, the observed variations in outcomes should be interpreted in the context of these factors. Adherence to the MD has the potential to improve survival rates and reduce the risk of CVDs in both the general population and individuals with a prior CVD. Further research is needed to explore the specific mechanisms underlying the protective effects of this dietary pattern and to better understand the role gender-related differences in its outcomes. Nevertheless, promoting the adoption of the MD could be an effective strategy for mitigating the burden of CVDs globally.

## 1. Introduction

Cardiovascular diseases (CVDs) are chronic disorders affecting the heart and circulatory system [1]. They typically develop gradually and can remain asymptomatic for extended periods [2]. CVDs currently stand as the leading cause of mortality worldwide, accounting for approximately one-third of global deaths [3]. In 2015 alone, over 400 million individuals were affected by CVDs, resulting in nearly 18 million deaths worldwide, representing 31% of total mortality [3]. The primary contributors to this statistic are coronary heart disease and cerebrovascular accidents (CVAs) [4]. For instance, in Spain alone, there were 253,091 reported deaths due to cardiovascular disease in 2020, as every reported by the National Institute of Statistics [5]. Given these concerning figures, promoting cardiovascular health should be a lifelong endeavor, commencing with the adoption of heart-healthy habits from an early age and continuing through old age [6].

Numerous factors contribute to the development of cardiovascular diseases, including non-modifiable ones such as genetic inheritance, sex, and age. Additionally, there are modifiable factors that primarily involve lifestyle behaviors like smoking, physical activity, and diet [2]. The latter set of factors holds significant importance as prevention strategies should primarily focus on modifying them. Preventive measures can be implemented at both the individual and population levels. Individual-level interventions typically emphasize the identification of risk factors and providing appropriate treatment for individuals classified as high-risk. In this approach, each person is treated individually [6]. However, such strategies should be complemented by interventions targeting the general population to reduce cardiovascular risk factors. Two primary intervention strategies can be discerned: health promotion and disease prevention [7]. Health promotion revolves around maintaining low cardiovascular risk, while disease prevention focuses on individuals at high risk of critical cardiovascular events [6]. Lifestyle modifications related to smoking, diet, and physical exercise play a pivotal role in cardiovascular disease prevention [8,9]. Diet, in particular, has been found to have a significant impact on cardiovascular disease control and the management of risk factors like obesity, high cholesterol, diabetes, and hypertension [10,11].

The Mediterranean Diet (MD) is recognized as a prevailing dietary pattern in several regions in the Mediterranean Basin, particularly Portugal, Spain, Italy, and Greece. Compliant with healthy nutritional recommendations, the MD entails a high consumption of fish, monounsaturated fats (mainly from olive oil), fruits, vegetables, whole grains, legumes, and nuts. Conversely, the MD is characterized by a low consumption of saturated fats, red meat, alcohol, and processed foods [12,13,14]. Notably, the MD has been associated with numerous health benefits, including protection against cardiovascular disease [15], reduced overall mortality [16], decreased risk of specific cancers [17,18], improved cognitive function [19,20], and mitigation of metabolic syndrome and its components, such as obesity, hypertension, hyperglycemia, and hyperlipidemia [11].

The body of evidence regarding the impact of the MD on the general population (primary prevention) is substantial, encompassing observational studies as well as clinical trials. However, recommendations for patients with a prior cardiovascular disease have primarily relied on observational evidence until recently, with the publication of the CORDIOPREV study [21]. Furthermore, multiple studies have explored the potential gender-related differences in the benefits of the MD concerning mortality and CVDs, with varying outcomes. Thus, the objective of this study is to provide an updated systematic review that examines the influence of the MD on mortality and CVDs, both in the general population and in patients with a prior CVD, while considering the potential impact of gender.

## 2. Materials and Methods

### 2.1. Search Question

The research question for this systematic review has been formulated using the PICO format (patient, intervention, control, outcome). The objective was to investigate the impact of implementing a Mediterranean diet on adverse cardiovascular outcomes, including all-cause cardiovascular mortality, myocardial infarction, angina, heart failure, and/or stroke. The review will focus on two distinct populations: a healthy population (primary prevention) and patients with a previous cardiovascular disease (secondary prevention).

### 2.2. Search Strategy

This systematic review follows the recommendations of the PRISMA 2020 Declaration [22]. The search for and selection of the studies included in the review were conducted by two independent researchers (A.L. and C.R.-P.), to comply with the peer review criteria. The following electronic databases were used: Medline, Scopus, and Web of Science. The search was limited to a period from 1995 to 2022 in all databases.

The inclusion criteria were as follows: (1) prospective cohort studies or randomized clinical trials; (2) adherence to a Mediterranean diet analyzed as exposure; (3) the outcome of interest included major cardiovascular adverse events in either the healthy population (primary prevention) or patients with previous cardiovascular disease (secondary prevention). Studies were selected if adherence to the Mediterranean diet was assessed by a specific questionnaire that evaluated the key components of the Mediterranean diet (number of servings every meal/week of cereals, fruits, vegetables, legumes, fish, poultry, white meat, red meat, olive oil and/or nuts). Studies were only included when incidence of cardiovascular disease, coronary heart disease, stroke, heart failure, or cardiovascular or all-cause mortality was reported, either as an absolute risk reduction, relative risk reduction, or hazard ratio.

The following search terms were used in different combinations: “Mediterranean diet”, “mediterranean pattern”, “cardiovascular disease”, “myocardial infarction”, “coronary heart disease”, “coronary artery disease”, “ischemic heart disease”, “stroke”, “survival”, and “mortality”.

### 2.3. Study Selection

The Mendeley ^®^ software, version 1.19.8, was used to manage the bibliography in an orderly fashion.

After contrasting and eliminating duplicate articles, the remaining articles were screened by title and abstract. After screening, a final selection was made on the basis of the eligibility criteria outlined above. This selection was made by two independent reviewers. The final articles that met these criteria were chosen by both reviewers through discussion and the support of a third reviewer to establish a consensus.

### 2.4. Risk of Bias Assessment

The Newcastle Ottawa scale was used to assess the quality of non-randomized studies in terms of patient selection, covariate adjustment, and outcome evaluation [23]. The Oxford quality scoring system or Jadad scoring [24], a procedure used to assess the methodological quality of a clinical trial using objective criteria, was used for randomized clinical trials to assess randomization, blinding, and withdrawals.

## 3. Results

### 3.1. Study Selection

**Figure 1:**
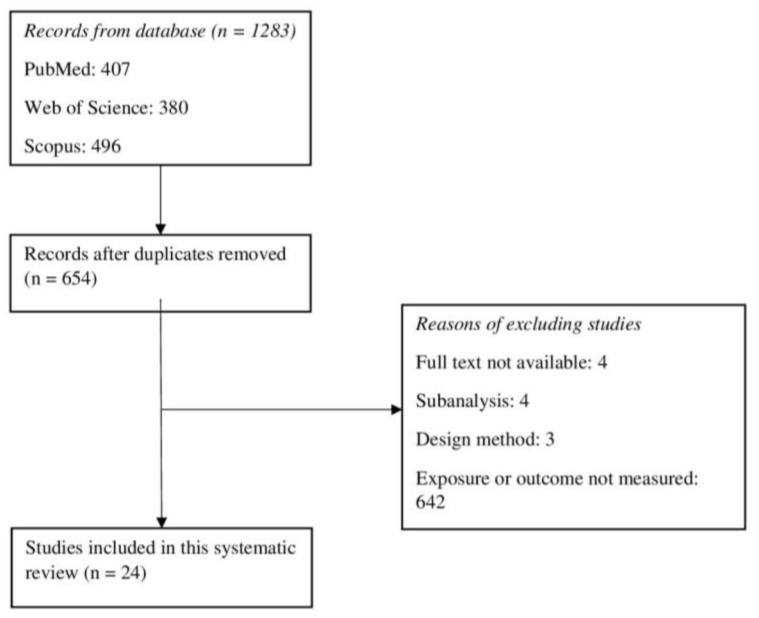
PRISMA flowchart of article selection.

A total of 1283 potential articles were selected. Duplicates were eliminated, and manuscripts were read by title and abstract; 622 articles were removed because they did not meet the inclusion criteria. For a second time, 32 full-text articles were reviewed by two researchers independently. Finally, the total sample that was selected following the inclusion and exclusion criteria included 24 studies. Although subanalysis was excluded from this review, four studies of two cohorts were included, as the outcomes were considered to be of interest. A PRISMA flowchart of the article selection can be seen in Figure 1.

### 3.2. Study Characteristics

The population included in this systematic review consisted of 721,113 healthy individuals and 39,304 patients with a prior cardiovascular disease. Fifteen studies, comprising fourteen prospective cohort studies and one randomized clinical trial, examined the impact of adherence to a Mediterranean diet on cardiovascular outcomes and mortality in the general population (primary prevention). Additionally, nine studies, consisting of seven prospective cohort trials and two randomized clinical trials, investigated this impact on patients who had had a previous cardiovascular disease (secondary prevention).

Table 1 and Table 2 present the key characteristics of the selected articles. To facilitate analysis, the studies will be assessed separately based on the population under study (general population—primary prevention and patients with a prior cardiovascular disease—secondary prevention), as well as considering gender differences.

In all cohort studies, dietary intake was assessed using a food frequency questionnaire or dietary history, which recorded the frequency of food consumption. Adherence to the Mediterranean diet was evaluated using various scoring systems that varied across studies. These scores assessed the frequency of intake of key nutritional components of the Mediterranean diet (e.g., vegetables, fruits, legumes, nuts, grains, fish, and seafood) as well as components considered detrimental to the diet (e.g., meat, poultry, dairy products, sweets). The Mediterranean Diet Score (MDS), proposed by Trichopoulou et al. [46,48], was the most commonly used scoring system. This scale assigns 0 points or 1 point based on the median intake of beneficial or detrimental components, respectively. Alcohol and fat intake are scored according to presumed beneficial standards and the ratio of monounsaturated to saturated lipids, respectively. The final score ranges from 0 to 9 points. Alternative scoring systems included the alternate MDS (aMDS) [49], which includes laver and kelp/sea mustard as vegetables and multigrain rice as a whole-grain product and excludes the ratio of monounsaturated fatty acid to saturated fatty acid and nuts, and the total and maximum score is seven components, with a higher score indicating a higher quality of the diet; the modified MDS (mMDS) [50], where the total mMDS score has a possible range of 0 (no conformity to a Mediterranean-style diet) to 42 (maximal conformity to a Mediterranean-style diet); the pyramid MDS (pyrMDS) [51] with 13 food groups, with a score of 0 to 10 for each group; the literature MDS (litMDS) [52]; and the rMED score [25], which includes nine components and a scoring range of 0 to 18 points.

The randomized clinical trials by Estruch et al. [27] and Delgado-Lista et al. [21] assessed adherence to the Mediterranean diet using periodic quantitative questionnaires containing 14 items and chemical biomarkers.

In some studies, adherence to the Mediterranean diet was analyzed as a categorical variable, comparing participants with intermediate and high scores to those with lower scores (reference category). Other studies treated adherence as a continuous variable and reported hazard ratios for a certain increase in the diet score. All studies conducted multivariate analyses, adjusting for potential confounding variables, and occasionally presented results from different statistical models. For this review, the results from models that included a higher number of adjustment variables (or the model considered as the reference by the authors) are presented. A more detailed analysis can be found in the Supplementary Materials.

### 3.3. Impact of Mediterranean Diet on Cardiovascular Outcomes and Death in the General Population (Primary Prevention)

The studies focusing on primary prevention included adult participants from European countries or the USA. Among these studies, three specifically included only women, and one provided sex-specific data, which will be analyzed separately.

All the studies were prospective in design, with a mean follow-up period ranging from 3.6 to 20 years. Ten studies had a mean or median follow-up duration longer than 10 years. The primary outcomes assessed in these studies included all-cause mortality, cardiovascular mortality, myocardial infarction, stroke, and cardiovascular disease. The specific characteristics of the selected studies can be found in Table 1.

#### 3.3.1. All-Cause Mortality

The impact of a Mediterranean diet on all-cause mortality was evaluated in six prospective cohort studies [26,31,34,37,38,39] and one randomized clinical trial [27]. These studies included a total of 113,737 patients, with 9751 reported events of mortality (Table 3).

In all the observational studies, patients with higher scores on Mediterranean diet scales showed a lower risk of all-cause mortality compared to those with lower scores. The reduction in risk ranged from an HR of 0.44 (IC 95% 0.24–0.79) to 0.79 (0.69–0.91). When considering adherence to the Mediterranean diet as a continuous variable, five studies [26,31,34,37,39] reported a significant decrease in the risk of all-cause mortality with increasing diet scores. However, one study involving 1080 patients [38] did not find a significant reduction in risk with an increase of 1 point in the modified Mediterranean Diet Score.

The randomized clinical trial conducted by Estruch et al. [27], which assigned patients to either a Mediterranean diet supplemented with either extra-virgin olive oil or nuts or a control diet with general advice to reduce dietary fat, did not demonstrate a significant reduction in the secondary outcome of all-cause mortality among patients in the Mediterranean diet group.

#### 3.3.2. Cardiovascular Mortality

Data on cardiovascular mortality were obtained from six prospective cohort studies [26,29,30,31,37,38] and one randomized clinical trial [27], involving a total of 113,435 patients and 3595 reported events (Table 4). It was observed that cardiovascular disease accounted for almost one-third (29.9%) of all deaths in these studies.

Among the four studies [26,30,31] that assessed adherence to the Mediterranean diet as a categorical variable, a significant reduction in cardiovascular mortality was found in patients with higher adherence scores compared to those with lower scores. The reduction in risk ranged from an HR of 0.44 (95% CI 0.30–0.66) to 0.71 (0.49–1.04). Furthermore, five out of six studies [26,29,30,37] that analyzed adherence to the Mediterranean diet as a continuous variable reported a significant decrease in the risk of cardiovascular death.

However, it is worth noting that the PREDIMED trial, which randomized patients to the Mediterranean diet group, did not demonstrate a reduction in the secondary outcome of cardiovascular mortality among the patients assigned to the Mediterranean diet group.

#### 3.3.3. Major Cardiovascular Events

Additional outcomes were analyzed separately, including cardiovascular disease and coronary artery disease incidence, coronary artery mortality, myocardial infarction, and stroke, yielding mixed results. Please refer to the Supplementary Materials for detailed information on these outcomes.

Three studies, consisting of two prospective cohort studies [25,30] and one randomized clinical trial [27], reported composite outcomes of major cardiovascular events as their primary outcomes (Table 5). Higher adherence to the Mediterranean diet was associated with a significant reduction (HR 0.65; CI95% 0.53–0.90) in the composite outcome of myocardial infarction, stroke, or cardiovascular disease mortality. Additionally, another study reported a significant reduction (HR 0.6; CI95% 0.47–0.77) in the composite outcome of myocardial infarction (fatal and non-fatal) and angina requiring revascularization.

In the PREDIMED trial, which focused on patients with a prior cardiovascular disease, the primary outcome was a composite of myocardial infarction, stroke, and cardiovascular disease. Patients randomized to the Mediterranean diet group demonstrated an HR of 0.72 (CI95% 0.54–0.96) in the composite outcome, with no significant differences observed between the variants of the Mediterranean diet (supplemented with extra-virgin olive oil or nuts). Secondary analysis of the individual components of the outcome revealed a significant reduction in the risk of stroke (HR 0.61; 95% CI: 0.44–0.86), while no significant differences were observed between the groups regarding all-cause mortality, cardiovascular mortality, or myocardial infarction risk.

### 3.4. Impact of Mediterranean Diet on Cardiovascular Outcomes and Death in Patients with a Prior Cardiovascular Disease (Secondary Prevention)

Nine studies, consisting of seven prospective cohort studies and two randomized clinical trials, were included in this systematic review. These studies focused on patients with a previous coronary artery disease and recruited a total of 39,304 participants from various European countries and the USA. The mean or median follow-up duration of these studies ranged from 3.7 to 10 years. The primary outcomes assessed in these studies were all-cause mortality, cardiovascular mortality, myocardial infarction, stroke, and cardiovascular disease. Table 2 provides an overview of the key characteristics of the selected studies, including information on study design, sample size, and outcomes assessed.

#### 3.4.1. All-Cause Mortality

All-cause mortality data were obtained from seven prospective cohort studies [40,41,42,43,44,45,47,48], which included a total of 38,881 patients and reported 7267 events. In all of these studies, higher adherence to the Mediterranean diet was significantly associated with a reduced risk of mortality (Table 6). Patients who had higher scores on the various Mediterranean diet scales exhibited a reduction in the risk of overall mortality ranging from an HR of 0.51 (CI95% 0.44–0.59) to 0.80 (0.67–0.95). Moreover, an increase of 2 points in the Mediterranean Diet Score was associated with a significant decrease in the risk of all-cause death. These findings indicate a consistent and significant protective effect of adherence to the Mediterranean diet against all-cause mortality across the included studies.

#### 3.4.2. Cardiovascular Mortality

Data on cardiovascular mortality were obtained from three prospective cohort studies [41,44,48], which included a total of 19,897 patients and reported 2024 cardiovascular events (Table 7). Among the fatal events analyzed, cardiovascular diseases accounted for 53% of the cases. In the studies that analyzed adherence to the Mediterranean dietary pattern as a categorical variable, no significant differences were found between patients with lower adherence and those with the highest adherence regarding cardiovascular mortality. However, two of the studies [41,48] reported that a 2-point increase in the Mediterranean Diet Score (MDS) was associated with a reduction in the risk of cardiovascular mortality. Conversely, an increase in the alternate MDS score did not show a significant reduction in cardiovascular mortality. These findings suggest that higher adherence to the Mediterranean diet may be associated with a lower risk of cardiovascular mortality, as indicated by the significant reduction observed with increasing MDS scores in two of the included studies.

#### 3.4.3. Composite Cardiovascular Outcomes

Two randomized trials [21,42] and one prospective cohort study [45] examined the composite outcomes of major cardiovascular events (Table 8). In the study by Shikany et al. [45], patients with higher adherence to the Mediterranean diet presented a reduction in the risk of myocardial infarction and coronary heart disease mortality compared to the patients with the lowest scores (HR 0.78; CI 95% 0.62–0.98). The clinical trial conducted by Delgado-Lista et al. [21], which randomized 1002 patients to either the Mediterranean diet or a low-fat diet, the HR for the composite outcome, including myocardial infarction, revascularization, stroke, peripheral artery disease, and cardiovascular death, was 0.75 (CI 95% 0.57–0.99). Additionally, the clinical trial by De Lorgeril et al. [42], which randomized 423 patients to either the Mediterranean diet or a control diet, demonstrated a substantial reduction in the composite outcome of myocardial infarction and cardiovascular death, as well as a in the composite outcome of myocardial infarction, cardiovascular death, unstable angina, heart failure, stroke, and embolism. These findings suggest that adherence to the Mediterranean diet may significantly lower the risk of experiencing major cardiovascular events.

Sex-related differences in the effect of the Mediterranean diet on cardiovascular outcomes and death.

Three studies included in this review specifically focused on healthy women [28,32,36], while the study by Mitrou et al. [35] presented data separately for both sexes without a pooled analysis. Additionally, ten studies included in this review provided sex-specific hazard ratios for the primary outcomes, and some conducted subgroup or sensitivity analyses. These studies encompassed a total of 688,533 patients, including 657,065 healthy participants and 31,468 patients with a prior cardiovascular disease. Among these patients, 280,980 were men and 470,533 were women.

The available data on sex-related differences in all-cause mortality and adherence to the Mediterranean diet were examined in eight prospective cohort studies (Table 9). The studies conducted by Buckland et al. [26], Mitrou et al. [35], and Trichopoulou et al. [39] demonstrated a significant reduction in all-cause mortality regardless of sex. However, in the study by Martínez-González et al. [34], the independent analysis of hazard ratios did not reveal a significant risk reduction in women (p-trend 0.41). Notably, the number of events was higher among men in all studies. Among the seven studies that reported the number of all-cause deaths by sex, comprising 477,669 patients (251,755 men and 225,914 women), there were 20,957 events in the men group compared to 9934 events in the women group.

The PREDIMED and CORDIOPREV randomized clinical trials conducted subgroup analyses based on sex for their primary outcomes. In the PREDIMED trial, which enrolled 3165 men and 4282 women, the analysis did not indicate a significant influence of sex on the effect of the Mediterranean diet on the composite outcome of myocardial infarction, stroke, and cardiovascular disease death (hazard ratio (HR) in men 0.73, CI 95% 0.5–1.07; HR in women 0.69, CI 95% 0.51–0.94; p for interaction 0.62). Conversely, in the CORDIOPREV trial, which included 827 men and 175 women with prior myocardial infarction, sex was considered a potential interaction factor in the relationship between Mediterranean diet and major cardiovascular events (HR for men 0.68, CI 95% 0.50–0.94; HR for women 1.27, CI 95% 0.64–2.49; p for interaction 0.03).

Furthermore, the studies conducted by Fung et al. [32], Lagiou et al. [32], and Tektonidis et al. [36] examined the impact of the Mediterranean diet on cardiovascular outcomes in healthy women. The study by Fung et al. [28] reported a significant reduction in the risk of coronary artery disease (CAD) and CAD-associated mortality in women with high adherence to the Mediterranean diet (HR for CAD mortality comparing the highest to the lowest quintile 0.58, CI 95% 0.45–0.75; for CAD 0.71, CI 95% 0.62–0.82). Additionally, the study by Tektonidis et al. [36] demonstrated that higher adherence to the Mediterranean diet was associated with a significant risk reduction in myocardial infarction (HR 0.92, CI 95% 0.89–0.96), ischemic stroke (HR 0.94, CI 95% 0.90–0.98), and heart failure (HR 0.94, CI 95% 0.91–0.97). However, in the study by Lagiou et al. [32], conducted among healthy women aged 30–49 years, the Mediterranean diet was not associated with a significant risk reduction in all-cause mortality in the fully adjusted model (HR 0.85, CI 95% 0.67–1.08).

### 3.5. Methodological Quality and Risk of Bias

The quality of the studies (randomized clinical trials) was assessed using the Newcastle–Ottawa scale for cohort studies and the Oxford quality scoring system for randomized clinical trials. The results of the analysis can be found in the Supplementary Materials.

## 4. Discussion

This systematic review provides an updated and comprehensive analysis of the impact of adherence to the Mediterranean diet on a wide range of cardiovascular outcomes and mortality. The findings from both observational studies and clinical trials indicate that higher adherence to the Mediterranean diet is associated with a reduced risk of all-cause mortality, cardiovascular disease, including myocardial infarction, stroke, and cardiovascular mortality.

Although some studies have reported differences in the benefits of the Mediterranean diet between men and women, these differences are likely due to lower event rates and a generally lower cardiovascular risk profile in women. Overall, adherence to the Mediterranean diet appears to reduce the risk of mortality and cardiovascular events in women.

The review includes analysis of the PREDIMED and CORDIOPREV trials, which investigated the impact of the Mediterranean diet on cardiovascular outcomes in the healthy population and in patients with a prior cardiovascular disease, respectively. In the PREDIMED trial, the Mediterranean diet showed a significant risk reduction in the composite outcome of myocardial infarction, stroke, and cardiovascular disease death. However, this reduction was mainly driven by a decrease in the risk of stroke, while no significant differences were found for myocardial infarction or cardiovascular disease death. The lower-than-expected event rate in the trial may have limited the statistical power to evaluate each endpoint individually.

Evidence regarding the benefits of the Mediterranean diet in patients with a prior cardiovascular disease has been more limited, with most studies based on observational data and one small clinical trial. However, high-quality studies with a substantial number of events have shown a comparable reduction in all-cause mortality risk for patients with a prior cardiovascular disease as seen in primary prevention. The evidence for cardiovascular mortality risk reduction in this population is conflicting, with some studies demonstrating benefits while others have not. Differences in the assessment of adherence to the Mediterranean diet [37,46,48] and the lower event rates in women may contribute to these discrepancies [53].

The CORDIOPREV trial randomized patients with prior coronary artery disease to follow either a Mediterranean diet or a low-fat diet. Patients in the Mediterranean diet group showed a significant risk reduction in the composite outcome of myocardial infarction, revascularization, stroke, peripheral artery disease, and cardiovascular death. This trial supports the existing evidence of the benefits of the Mediterranean diet in patients following an acute cardiovascular event, and emphasizes the importance of promoting high adherence to the diet in this population.

The influence of sex on the potential benefits of the Mediterranean diet in cardiovascular health has been a topic of debate. The review provides a structured analysis of outcomes based on sex and reveals that most studies analyzing the impact of sex and the Mediterranean diet showed a significant reduction in all-cause mortality in women [26,35,39]. However, the number of events in women was generally lower [32] compared that in men, which may have influenced the results. More studies that accurately represent female patients are needed to further understand the potential sex-specific effects of the Mediterranean diet. Nevertheless, based on the wide body of evidence supporting the recommendation of the Mediterranean diet for the general population and patients with cardiovascular diseases, women should receive the same dietary recommendations as men.

To the best of our knowledge, this systematic review is the most extensive and up-to-date analysis to date on the association between the Mediterranean diet and all-cause mortality and cardiovascular disease, with a focus on sex-specific analysis. However, there are limitations to consider, including the possibility of missing relevant articles in the initial search, variations in exposure and outcome definitions among the screened articles, potential residual confounding in observational studies, and heterogeneity among studies in terms of diet assessment, study regions, age groups, cardiovascular risk profiles, and history of cardiovascular disease. These limitations may affect the interpretation and comparability of the results.

## 5. Conclusions

This systematic review offers an up-to-date and comprehensive analysis of the evidence supporting the potential benefits of adhering to the Mediterranean diet in terms of improved survival and reduced risk of cardiovascular disease. The findings indicate that both the general population and individuals with a prior cardiovascular disease may experience positive effects from following a Mediterranean diet. Although there are suggestions of potential benefits for women, it is important to note that more randomized clinical trials with adequate representation of female participants are needed to provide stronger evidence and better understand the specific effects in women.

## Figures and Tables

**Table 1:** Study characteristics of articles that analyzed the impact of Mediterranean diet in cardiovascular outcomes and death in the general population.

Author (Year)	Participants n, Age (Years)	Country	MD Assessment	Design and Follow-Up	Outcomes
Buckland G et al., (2009) [25]	41,078 29–69 (range); 49.3 (mean)	Spain	rMED (0–18)	Prospective cohort 10.4 years (mean)	Fatal and non-fatal MI, angina requiring revascularization
Buckland G et al., (2011) [26]	40,622 29–69 (range); 49.3 (mean)	Spain	rMED (0–18)	Prospective cohort 13.4 years (mean)	All-cause and cardiovascular mortality
Estruch R et al., (2013) [27]	7447 participants with high CVD risk 50–80 (range); 67 (mean)	Spain	Quantitative score (0–14) Biomarkers	RCT (MD with EVOO/nuts vs. control) 4.8 years (median)	Composite of MI, stroke, and cardiovascular mortality
Fung TT et al., (2009) [28]	74,886 women 38–63 (range)	USA	Alternate MDS	Prospective cohort 20 years	CAD mortality, CAD
Gardener H et al., (2011) [29]	2568 >40; 68.6 (mean)	USA	MDS (0–9)	Prospective cohort 9 years	Composite of ischemic stroke, MI, or cardiovascular mortality
Hoevenaar-Blom MP et al., (2012) [30]	34,708 20–70 (range)	Netherlands	MDS (0–9)	Prospective cohort 11.8 years (mean)	Cardiovascular mortality, CVD, MI, and stroke
Knoops KTB et al., (2004) [31]	2339 70–90 (range)	Europe	mMDS (0–8)	Prospective cohort 10 years	All-cause, CVD and CAD mortality
Lagiou P et al., (2006) [32]	42,237 women 30–49 (range)	Sweden	MDS (0–9)	Prospective cohort 12 years	All-cause mortality
Martínez-González MA et al., (2011) [33]	13,609 38 (mean)	Spain	MDS (0–9)	Prospective cohort 4.9 years	Cardiovascular disease
Martínez-González MA et al., (2012) [34]	15,535 38 (mean)	Spain	MDS (0–9)	Prospective cohort 6.8 years	All-cause mortality
Mitrou PN et al., (2007) [35]	380,296 50–71 (range)	USA	aMDS (0–9)	Prospective cohort 10 years	All-cause and CVD mortality
Tektonidis TG et al., (2015) [36]	32,921 women 48–83 (range)	Sweden	mMDS (0–8)	Prospective cohort 10 years	MI, heart failure and stroke
Tong TYN et al., (2016) [37]	23,902 40–79 (range)	UK	tMDS (0–18) MDS (0–9) Others	Prospective cohort 17 years	Cardiovascular mortality, CVD
Tognon G et al., (2013) [38]	1849	Denmark	mMDS (0–8)	Prospective cohort	All-cause mortality, cardiovascular mortality, CVD, MI
Trichopoulou A et al., (2003) [39]	41,078 29–69 (range); 49.3 (mean)	Greece	MDS (0–9)	Prospective cohort 44 months (median)	All-cause and cardiovascular mortality

CAD: coronary artery disease. CVD: cardiovascular disease. MD: Mediterranean diet. MI: myocardial infarction.

**Table 2:** Study characteristics of articles that analyzed the impact of Mediterranean diet in cardiovascular outcomes and death in the general population.

Author (Year)	Participants n, Age (Years)	Country	MD Assessment	Design and Follow-Up	Outcomes
Barzi F et al., (2003) [40]	11,323 patients with MI 19–90 (range); 59.4 (mean)	Italy	Overall nutrition score 0–10	Prospective cohort 6.5 years	All-cause mortality
Bonaccio M et al., (2018) [41]	1180 patients with prior CVD ≥35; 67.7 (mean)	Italy	MDS (0–9)	Prospective cohort 7.9 years	All-cause mortality, cardiovascular and CAD mortality
Delgado-Lista J et al., (2022) [21]	1002 patients with previous CAD (ACS or high-risk CAD)	Spain	Quantitative score 0–14	RCT (MD vs. low-fat diet) 7 years	Composite of MI, revascularization, stroke, peripheral artery disease, and CVD death
De Lorgeril M et al., (1999) [42]	423 patients < 70 years with prior MI	France	NA	RCT (MD vs. control) 46 months	Composite outcome CVD death, MI, angina, stroke, heart failure, pulmonary, or peripheral embolism
Iestra J et al., (2006) [43]	426 patients with prior MI ≥70	Europe	MDS (0–9)	Prospective cohort 10 years	All-cause mortality
Lopez-Garcia E et al., (2014) [44]	17,415 participants with prior MI, stroke, angina, bypass or angioplasty 40–75 (range); 69 (mean)	USA	Alternate MDS	Prospective cohort 7.7 years (median)	All-cause mortality, cardiovascular mortality
Shikany JM et al., (2018) [45]	3562 participants with previous CAD, ≥45; 68.9 (mean)	USA	MDS (0–9)	Prospective cohort 7.1 years	All-cause mortality, CAD (MI and CAD death)
Trichopoulou A et al., (2005) [46]	1302 participants with CAD 20–86 (range)	Greece	MDS (0–9)	Prospective cohort 3.78 years	All-cause mortality, cardiovascular mortality
Trichopoulou A et al., (2007) [47]	2671 patients with previous MI >60	Europe	MDS (0–9)	Prospective cohort 6.7 years	All-cause mortality

ACS: acute coronary syndrome. CAD: coronary artery disease. CVD: cardiovascular disease. MD: Mediterranean Diet. MI: myocardial infarction. RCT: randomized clinical trial.

**Table 3:** Studies that analyzed risk of all-cause mortality and adherence to Mediterranean diet in the general population.

**Prospective Cohort Studies**				
**Author (Year)**	**Score**	**Medium vs. Low**	**High vs. Low**	**Per Point Increase**
Buckland G et al., (2011) [26]	rMED	0.88 (0.79–0.99)	0.79 (0.69–0.91)	0.94 (0.9–0.97) every 2 points
Martínez-González MA et al., (2012) [34]	MDS	0.66 (0.39–1.11)	0.44 (0.24–0.79)	0.76 (0.61–0.94) every 2 points
Knoops KTB et al., (2004) [31]	mMDS	NA	0.77 (0.68–0.88)	NA
Tong TYN, et al., (2016) [37]	rMED MDS	NA NA	NA NA	0.97 (0.94–0.99) per SD 0.96 (0.93–0.98) per SD
Tognon G, et al., (2013) [38]	mMDS	NA	NA	0.95 (0.91–1.00) per point
Trichopoulou A et al., (2003) [39]	MDS	NA	NA	0.75 (0.64–0.87) every 2 points
**Randomized Clinical Trial**				
**Author (Year)**		**MD with EVOO**	**MD with Nuts**	**Combined MD**
Estruch R et al., (2013) [27]		0.82 (0.64–1.07)	0.97 (0.74–1.26)	0.89 (0.71–1.12)

EVOO: extra-virgin olive oil. MD: Mediterranean diet. NA: Not Applicable. SD: standard deviation.

**Table 4:** Studies that analyzed risk of cardiovascular mortality and adherence to Mediterranean diet in the general population.

**Prospective Cohort Studies**				
**Author (Year)**	**Score**	**Medium vs. Low Risk**	**High vs. Low Risk**	**Per Point Increase**
Buckland G et al., (2011) [34]	rMED	0.84 (0.66–1.09)	0.66 (0.49–0.89)	0.88 (0.81–0.95) every 2 points
Gardener H et al., (2011) [29]	MDS	4p: 0.70 (0.48–1.02) 5p: 0.69 (0.47–1.00)	0.71 (0.49–1.04)	0.91 (0.85–0.98) per point
Hoevenaar-Blom MP et al., (2012) [30]	MDS	0.60 (0.44–0.80)	0.44 (0.30–0.66)	0.78 (0.69–0.88) per point
Knoops KTB et al., (2004) [31]	mMDS	NA	0.71 (0.58–0.88)	NA
Tong TYN, et al., (2016) [37]	rMED MDS	NA	NA	0.94 (0.90–0.99) per SD 0.94 (0.89–0.98) per SD
Tognon G, et al., (2013) [38]	mMDS	NA	NA	0.96 (0.89–1.05) per unit
**Randomized Clinical Trial**				
**Author (Year)**		**MD with EVOO**	**MD with Nuts**	**Combined MD**
Estruch R et al., (2013) [26]		0.69 (0.41–1.16)	1.01 (0.61–1.66)	0.83 (0.54–1.29)

EVOO: extra-virgin olive oil. MD: Mediterranean diet. NA: Not Applicable. SD: standard deviation. 4–5p: 4 or 5 points on the MDS scale.

**Table 5:** Composite outcomes of major cardiovascular events and adherence to Mediterranean diet in the general population.

**Prospective Cohort Studies**				
**Author (Year)**	**Score**	**Medium vs. Low**	**High vs. Low**	**Per Point Increase**
Buckland G et al., (2009) [25] Fatal and non-fatal MI, angina requiring revascularization	rMED	0.86 (0.7–1.04)	0.6 (0.47–0.77)	0.94 (0.91–0.97)
Hoevenaar-Blom MP et al., (2012) [30] MI, stroke, CVD death	MDS	0.72 (0.61–0.85)	0.65 (0.53–0.80)	0.85 (0.80–0.91) per point
**Randomized Clinical Trial**				
**Author (Year)**		**MD with EVOO**	**MD with Nuts**	**Combined MD**
Estruch R et al., (2013) [27] MI, stroke, CVD death		0.70 (0.54–0.92)	0.72 (0.54–0.96)	0.71 (0.56–0.90)

CVD: cardiovascular disease. EVOO: extra-virgin olive oil. MD: Mediterranean diet. MI: myocardial infarction.

**Table 6:** Studies that analyzed risk of all-cause mortality and adherence to Mediterranean diet in patients with a prior cardiovascular disease.

Author (Year)	Score	Medium vs. Low	High vs. Low	Per Point Increase
Barzi F et al., (2003) [40]	Other	0.69 (0.61–0.79)	0.51 (0.44–0.59)	0.85 (0.82–0.88) per point
Bonaccio M et al., (2018) [41]	MDS	0.82 (0.60–1.12)	0.69 (0.47–0.99)	0.84 (0.70–1.00) every 2 points
Iestra J et al., (2006) [43]	MDS	NA	0.75 (0.57–0.97)	NA
Lopez-Garcia E et al., (2014) [44]	aMDS	Q2: 0.98 (0.89, 1.08) Q3: 0.90 (0.81, 1.00)	Q4: 0.94 (0.81, 1.08) Q5: 0.81 (0.72–0.91)	0.93 (0.89, 0.97) every 2 points
Shikany JM et al., (2018) [45]	MDS	0.98 (0.85–1.13)	0.80 (0.67–0.95)	NA
Trichopoulou A et al., (2005) [48]	MDS	NA	NA	0.73 (0.58–0.93) every 2 points
Trichopoulou A et al., (2007) [47]	MDS	NA	NA	0.82 (0.73–0.93) every 2 points

MD: Mediterranean diet. NA: Not Applicable. Q2-Q5: second to fifth quintiles.

**Table 7:** Studies that analyzed risk of cardiovascular mortality and adherence to Mediterranean diet in patients with a prior cardiovascular disease.

Prospective Cohort Studies				
Author (Year)	Score	Medium vs. Low	High vs. Low	Per Point Increase
Bonaccio M et al., (2018) [41]	MDS	0.73 (0.48–1.12)	0.61 (0.35–1.01)	0.77 (0.61–0.97) every 2 points
Lopez-Garcia E et al., (2014) [44]	aMDS	Q2: 1.00 (0.79, 1.27) Q3: 0.99 (0.78, 1.25)	Q4: 1.02 (0.87, 1.21) Q5: 0.85 (0.67, 1.09)	0.96 (0.89, 1.04) every 2 points
Trichopoulou A et al., (2005) [48]	MDS	NA	NA	0.69 (0.52–0.93) every 2 points

Major Q2–Q5: second to fifth quintiles.

**Table 8:** Composite outcomes of major cardiovascular events and adherence to Mediterranean diet in patients with a prior cardiovascular disease.

**Prospective Cohort Studies**			
**Author (Year)**	**Score**	**Medium vs. Low**	**High vs. Low**
Shikany JM et al., (2018) [45] MI, CAD mortality	MDS	0.91 (0.76–1.10)	0.78 (0.62–0.98)
**RCT**			
**Author (Year)**	**Mediterranean Diet vs. Control Diet**		
Delgado-Lista J et al., (2022) [21] MI, revascularization, stroke, peripheral artery disease, and cardiovascular death	HR (7 models) ranged from 0.719 (95% CI 0.541–0.957) to 0.753 (0.568–0.998)		
De Lorgeril M et al., (1999) [42]	CO 1 (MI, CVD death): 0.23 (0.11–0.48) CO 2 (MI, CVD death, unstable angina, HF, stroke, embolism): 0.30 (0.18–0.51)		

CAD: coronary artery disease. CO: composite outcome. CVD: cardiovascular disease. HF: heart failure. MI: myocardial infarction.

**Table 9:** Analysis of sex differences in adherence to Mediterranean diet and all-cause mortality.

**Primary Prevention**					
**Author (Year)**		**Medium vs. Low**	**High vs. Low**	**Per Point Increase**	***p***
Buckland G et al., (2011) [26]	Men (15,324)	0.88 (0.75–1.02)	0.76 (0.63–0.9)	-	p-interaction: 0.512
	Women (25,298)	0.89 (0.75–1.06)	0.85 (0.68–1.06)	-	
Martínez-González MA et al., (2012) [34]	Men (6271)	-	-	0.67 (0.51–0.88) every 2 points	p-trend 0.004
	Women (9264)	-	-	0.83 (0.53–1.29) every 2 points	p-trend 0.41
Mitrou PN et al., (2007) [35]	Men (214 284)	0.91 (0.88–0.94)	0.79 (0.76–0.83)	-	-
	Women (166 012)	0.89 (0.85–0.93)	0.80 (0.75–0.85)	-	-
Trichopoulou A et al., (2003) [39]	Men (8895)	-	-	0.78 (0.65–0.94) every 2 points	-
	Women (13,148)	-	-	0.69 (0.53–0.90) every 2 points	-
**Secondary Prevention**					
Barzi F et al., (2003) [40]	Men (9625)	-	-	0.86 (0.83–0.89) per point	p-interaction: 0.13
	Women (1698)	-	-	0.81 (0.74–0.87) per point	
Iestra J et al., (2006) [43]	Men (284)	-	0.74 (0.55–1.00)	-	-
	Women (142)	-	0.86 (0.46–1.61)	-	-
López-García E et al., (2014) [44]	Men (6137)	0.85 (0.74, 0.98) *	0.79 (0.68, 0.91) *	0.92 (0.86, 0.97) every 2 points	p-trend: 0.003
	Women (11,278)	0.95 (0.81, 1.11) *	0.85 (0.71, 1.02) *	0.95 (0.88, 1.01) every 2 points	p-trend: 0.11
Trichopoulou A et al., (2005) [48]	Men (726)			0.69 (0.53–0.90) every 2 points	p-interaction: 0.33
	Women (576)			0.89 (0.56–1.41) every 2 points	

* Hazard ratio included in the medium adherence group corresponds to third quintile; high adherence corresponds to fifth quintile.

## Data Availability

Data sharing not applicable.

## Supplementary Materials

The following supporting information can be downloaded at: https://www.mdpi.com/article/10.3390/nu15153356/s1, Table S1: Different scores used to assess adherence to Mediterranean Diet in the studies included in the analysis, Table S2: Food ítems to evaluate adherence to a Mediterranean diet and example of scoring system in the PyrMDS score, Table S3: Pyramid based Mediterranean diet score (PyrMDS) scoring criteria.
