# Supplementary material for: Mediterranean Diet for Primary and Secondary Prevention of Cardiovascular Disease and Mortality: An Updated Systematic Review
Source: Nutrients. 2023 Jul 28;15(15):3356. doi: 10.3390/nu15153356 (PMC10421390; doi:10.3390/nu15153356)
Supplement: Supplementary file 1 [file nutrients-15-03356-s001.zip › nutrients-2519997-supplementary.pdf]

Table S1. Different scores used to assess adherence to Mediterranean Diet in the studies included in the analysis.

rMED score<br><br>Buckland et al. 2009 [25]	rMED component (apart from alcohol) was measured as grams per 1,000 kcal/day (to express intake as energy density) and was dividedinto tertiles of dietary intake. A value of 0, 1, and 2 was assigned to the first, second, and third tertiles of intake, respectively, positively scoring higher intakes for the 6 components presumed to fit the Mediterranean diet: fruit (including nuts and seeds but excluding fruit juices), vegetables (excluding potatoes), legumes, cereals (including whole-grain and refined flour, pasta, rice, other grains, and bread (69.5% of total cereals)), fresh fish (including seafood), and olive oil. The scoring was reversed for 2 components presumed not to fit the Mediterranean diet: total meat (including processed meat) and dairy products (including low-fat and high-fat milk, yogurt, cheese, cream desserts, and dairy and nondairy creams), positively scoring lower intakes. Alcohol, considered beneficial in moderation, was scored as a dichotomous variable by using the same ranges defined in previous EPIC studies. Two points were assigned for moderate consumers (5–25 g/day for women and 10–50 g/day for men) and 0 points for above and below the sex-specific range.	Low score 0-6<br>Medium score 7-10<br>High score 11-18
Estruch et al. [27]	Quantitative questionnaire<br>1 Do you use olive oil as main culinary fat? Yes<br>2 How much olive oil do you consume in a given day (including oil used for frying, salads, out of house meals, etc.)? 4 or more tablespoons<br>3 How many vegetable servings do you consume per day? (1 serving = 200g - consider side dishes as 1/2 serving) 2 or more (at least 1 portion raw or as salad)<br>4 How many fruit units (including natural fruit juices) do you consume per day? 3 or more<br>5 How many servings of red meat, hamburger, or meat products (ham, sausage, etc.) do you consume per day? (1 serving = 100-150 g) Less than 1<br>6 How many servings of butter, margarine, or cream do you consume per day? (1 serving = 12 g) Less than 1<br>7 How many sweet/carbonated beverages do you drink per day? Less than 1<br>8 How much wine do you drink per week? 7 or more glasses<br>9 How many servings of legumes do you consume per week? (1 serving = 150 g) 3 or more<br>10 How many servings of fish or shellfish do you consume per week? (1 serving: 100-150 g fish, or 4-5 units or 200 g shellfish) 3 or more<br>11 How many times per week do you consume commercial sweets or pastries (not homemade), such as cakes, cookies, biscuits, or custard? Less than 3<br>12 How many servings of nuts (including peanuts) do you consume per week? (1 serving = 30 g) 3 or more<br>13 Do you preferentially consume chicken, turkey or rabbit meat instead of veal, pork, hamburger or sausage? Yes<br>14 How many times per week do you consume vegetables, pasta, rice, or other dishes seasoned with sofrito (sauce made with tomato and onion, leek, or garlic, simmered with olive oil)? 2 or more	
MDS<br><br>Trichopoulou et al.1995 [46]	For beneficial components (vegetables, legumes, fruits and nuts, cereal, and fish), persons whose consumption was below the median were assigned a value of 0, and persons whose consumption was at or above the median were assigned a value of 1. For components presumed to be detrimental (meat, poultry, and dairy products, which are rarely nonfat or low-fat in Greece), persons whose consumption was below the median were assigned a value of 1, and persons whose consumption was at or above the median were assigned a value of 0. For ethanol, a value of 1 was assigned to men who consumed between 10 and 50 g per day and to women who consumed between 5 and 25 g per day. Finally, for fat intake, we used the ratio of monounsaturated lipids to saturated lipids, rather than the ratio of polyunsaturated to saturated lipids, because in Greece, monounsaturated lipids are used in much higher quantities than polyunsaturated lipids. Thus, the total Mediterranean-diet score ranged from 0 (minimal adherence	Low score 0-3<br>Medium score 4-5<br>High score 6-9

	to the traditional Mediterranean diet) to 9 (maximal adherence).	
aMed score<br><br>Kim et al., 2011 [49]	A standard portion size and 9 possible frequency of consumption responses, ranging from "never or less than once per month" to "_6 times per day" were given for each food. Total energy and nutrient intake was calculated by summing energy or nutrients from all foods.<br>The aMed score was adapted from the Mediterranean diet scale by Trichopoulou et al. Our components include vegetables (excluding potatoes), fruits, nuts, whole grains, legumes, fish, ratio of monounsaturated to saturated fat, red and processed meats, and alcohol.<br>Participants with intake above the median intake received 1 point for these categories; otherwise, they received 0 points. Red and processed meat consumption below the median received 1 point. We assigned 1 point for alcohol intake between 5 and 15 g/d. This represents approximately one 12-oz can of regular beer, 5 oz of wine, or 1.5 oz of liquor. The possible score range for aMed was 0 to 9, with a higher score representing closer resemblance to the Mediterranean diet.	Quintiles
mMDS<br><br>Trichopoulou et al., 2005 [50]	The modified Mediterranean diet score comprised 8 components: ratio of monounsaturated to saturated fat; legumes, nuts, and seeds; grains; fruit; vegetables and potatoes; meat and meat products; dairy products; and fish. Intake of each component was adjusted to daily intakes of 2500 kcal (10.5 MJ) for men and 2000 kcal (8.5 MJ) for women. The sexspecific median intake values were taken as cutoff points. The diet score varied from 0 (low-quality diet) to 8 (highquality diet). For the components monounsaturated fatty acids to saturated fatty acids (MUFA to SAFA) ratio; fruits and fruit products; vegetables and potatoes; legumes, nuts, and seeds; fish; and grains, a value of 1 was assigned to persons whose consumptionwas at least as high as the sex-specific median value, and 0 to the others. The vegetables group of the original Mediterranean diet score was replaced by the vegetables and potatoes group because the European classification system (EUROCODE) was used when the 2 food groups were assessed together.16 For meat and meat products and dairy products, a value of 1 was assigned to persons whose consumption was less than the sex-specific median and 0 to the others.	4 points or higher
pyrMDS<br><br>Rumawas et al., 2009 [51]	For PyrMDS, fifteen components were derived based on the pyramid, for which continuous scores of 0 to 1 were assigned for each component according to the participant's degree of adherence to the recommendation. For components for which high consumption was recommended (vegetables, legumes, fish), 0 to 1 points were assigned proportionally from no consumption to the recommended level of consumption. This scoring was reversed for components for which low consumption was recommended (red meat, processed meat, potato, sweets). For components for which moderate consumption was recommended (fruits, cereals, nuts, eggs, dairy, white meat), we assigned a score of 1 for consumption within the recommendation levels and 0 for no consumption. Overconsumption two-fold higher than the mid-point of the recommended intake was penalised and received a maximum of 0.5 point. For alcohol, sex-specific cut-offs were determined and moderate consumption was given 1 point; no consumption, 0.5 point; and overconsumption, 0 point.	
litMDS<br><br>Sofi et al., 2017 [52]	Sofi *et al.* developed a MDS based on literature (LitMDS), after systematically reviewing published cohort studies. The authors estimated weighted median intakes of nine Mediterranean diet components (vegetables, legumes, fruit and nuts, cereals, dairy, fish, meat, alcohol and olive oil) in different cohorts, and determined absolute cut-off points (with the exception of olive oil) to assign scores of 0, 1 or 2 for each component. For olive oil, the authors suggested 0 point for occasional use, 1 for frequent use and 2 for regular use, and we assigned 0 to all non-consumers, 1 for below median and 2 for above median levels of olive oil intake.	

Table S2. Food ítems to evaluate adherence to a Mediterranean diet and example of scoring system in the PyrMDS score.

Food groups	Items listed in the Food Frequency Questionnaire	Mediterranean diet score (score range)[1]			
		PyrMDS, based on the Mediterranean diet pyramid (0-15)	LitMDS, based on published literature (0-18)	mMDS, based on medians of dietary intakes (0-9)	tMDS, based on tertiles of dietary intakes (0-18)
Vegetables	Carrots, spinach, broccoli/spring greens/kale, brussels sprouts, cabbage, marrow/courgettes, cauliflower, parsnips/turnips/swedes, leeks, onions, garlic, mushrooms, sweet peppers, green salad/lettuce/cucumber/celery, watercress, tomatoes, sweetcorn, beetroot, coleslaw, avocado, vegetable soups, ketchup, pickles	0 to 1	0, 1, or 2	0 or 1	0, 1, or 2
Legumes	Peas, green beans/broad beans/runner beans, beansprouts, baked beans, dried lentils/beans/peas, tofu/soya meat/TVP/vegeburger	0 to 1	0, 1, or 2	0 or 1	0, 1, or 2
Fruits	Apples, pears, oranges/satsumas/mandarins, grapefruits, bananas, grapes, melon, peaches/plums/apricots, strawberries/raspberries/kiwi, tinned fruits, dried fruits,	0 to 1	0, 1, or 2	0 or 1	0, 1, or 2
Nuts	Peanuts and other nuts, peanut butter	0 to 1			
Cereals	Brown bread and rolls, wholemeal bread and rolls, crispbread, porridge/readybrek, breakfast cereal, brown rice, wholemeal pasta, white bread and rolls, cream crackers/cheese biscuits, white rice, white or green pasta, lasagne/moussaka, pizza	0 to 1	0, 1, or 2	0 or 1	0, 1, or 2
Dairy	Single or sour cream, double or clotted cream, low fat yoghurt/fromage frais, full fat or Greek yoghurt, dairy desserts, cheese, cottage cheese, milk	0 to 1	0, 1, or 2	0 or 1	0, 1, or 2
Fish	Fried fish, fish fingers/fish cakes, other white fish, oily fish, shellfish, fish roe/taramasalata	0 to 1	0, 1, or 2	0 or 1	0, 1, or 2
Red meats	Beef, pork, lamb, beefburgers, meat soups (if red meats e.g. oxtail soup)	0 to 1	0, 1, or 2	0 or 1	0, 1, or 2
Processed meats	Bacon, ham, corned beef, sausages, savoury pie, liver, liver pate and liver sausage	0 to 1			
White meats	Chicken and other poultry, meat soups (if white meats e.g. chicken soup)	0 to 1			
Eggs	Eggs, quiche	0 to 1			
Potatoes	Boiled/mashed/instant/jacket potatoes, chips, roast potatoes, potato salad	0 to 1			
Alcohol/Ethanol	Wine, beer/lager/cider, port/sherry/vermouth/liqueurs, spirits	0 to 1	0, 1, or 2	0 or 1	0, 1, or 2
Sweets	Sweet biscuits (chocolate), sweet biscuits (plain), cakes (homemade/ready made), buns/pastries cakes (homemade/ready made), fruits pies etc (homemade/ready made), sponge puddings (homemade/ready made), milk puddings, ice cream, chocolate, chocolate snacks, sweets/toffees/mints, sugar added to tea/coffee/cereal, jam/marmalade/honey, low cal/diet fizzy drinks, fizzy soft drinks, fruit squash/cordial	0 to 1			
Fat	Olive oil, monounsaturated fatty acids, polyunsaturated fatty acids, saturated fatty acids	0 to 1	0, 1, or 2	0 or 1	0, 1, or 2

Table S3: Pyramid based Mediterranean diet score (PyrMDS) scoring criteria.

Component	Recommended intake[1]	Score of 0[1]	Score of 1[1]
Vegetables	≥6/d	0/d	≥6/d
Legumes	≥2/wk	0/wk	≥2/wk
Fruits	3-6/d	0/d	3-6/d
Nuts	1-2/d	0/d	1-2/d
Cereals	3-6/d	0/d	3-6/d
Dairy	2/d	0/d	1.5-2.5/d
Fish	≥2/wk	0/wk	≥2/wk
Red meat	<2/wk	≥4/wk	<2/wk
Processed meat	≤1/wk	≥2/wk	≤1/wk
White meat	2/wk	0/wk	1.5-2.5/wk
Egg	2-4/wk	0/wk	2-4/wk
Potato	≤3/wk	≥6/wk	≤3/wk
Sweets	≤2/wk	≥4/wk	≤2/wk
Alcohol	2/d for men, 1/d for women	≥4/d for men, ≥2/d for women	1.5-2.5/d for men, 0.5-1.5/d for women
Olive oil	Principal source of dietary lipids	Non-consumers	Consumers
